# Assessing microclimatic influences in Colombo metropolitan area (CMA) amidst global climate change: a comprehensive study from 1980 to 2022

**DOI:** 10.1007/s10661-025-13648-9

**Published:** 2025-01-27

**Authors:** Panchali U. Fonseka, Hongsheng Zhang, Ranjith Premasiri, Chaminda Samarasuriya, Upaka Rathnayake

**Affiliations:** 1 Department of Earth Resource Engineering, Faculty of the Engineering, University of Moratuwa, Katubedda, 10400 Sri Lanka; 2 Arthur C Clarke Institute for Modern Technologies, Katubedda, 10400 Moratuwa Sri Lanka; 3 Department of Geography, The University of Hong Kong, Centennial Campus, Pokfulam Road, Hong Kong, China; 4 Department of Civil Engineering and Construction, Faculty of Engineering and Design, Atlantic Technological University, Sligo, F91 YW50 Ireland

**Keywords:** Climate change, Heat wave magnitude index, Rainfall anomaly index, Resilience, Satellite products, Seasonality index

## Abstract

Climate change has become an emerging topic, leading to widespread damage. However, when considering climate, attention is drawn to various scales, and urban microclimate has emerged as a trending subject due to its direct relevance to human living environments. Among the microclimatic factors, temperature and precipitation are utilized in order to identify trends. The identification of changes in precipitation and temperature from ground stations poses difficulties due to the lack of well-distributed stations; thus, satellite-based products are gaining popularity. The satellite products were validated against ground data, following which time-series and spatial analyses were conducted. The rainfall anomaly index, seasonality index, heat wave magnitude index, and mean temperature differ in the Colombo Metropolitan Area compared to the entire country. Each index is calculated decadal-wise to identify trends. By utilizing four climate indices, the analysis endeavors to investigate the microclimate identification in Colombo Metropolitan Area compared to its surrounding areas such as the Western Province and the entire country. This study aids local authorities in mitigating climate change by enhancing city resilience. These findings underscore the importance of understanding and addressing the impacts of climate change on temperature extremes to mitigate potential adverse effects on human activities and the environment. Understanding the specific reasons for spatial changes in rainfall anomalies often necessitates extensive climate modeling and data analysis.

## Introduction

Globally, climate change is being observed, and an increasing number of evidence are observed around the world (IPCC, 2022). Throughout the world, various forms of natural disasters such as a rise in temperature and sea level, droughts, floods, hurricanes, and landslides are happening as an indication of change in climate (Hussain et al., 2020). As per the Intergovernmental Panel on Climate Change (IPCC), the clarification of climate change is given as "change in the state of the climate that can be identified by changes in the mean and/or the variability of its properties and that persists for an extended period, typically decades or longer," and it is further noted that, even after the implementation of mitigation strategies, the climate continues to undergo shifts (IPCC, 2022). The major effects of climate change on human well-being are being experienced, and harm will continue to be caused in the future (Manning & Clayton, 2018).

During the past few decades, numerous studies have been conducted to investigate rainfall and temperature variability globally, regionally, and subregionally (Alizadeh, 2023; Azamathulla et al., 2018; Fathian et al., 2020; Lipczynska-Kochany, 2018; Loo et al., 2015; Ma et al., 2021). South Asia is characterized by trends in climate and its variability (Srivastava, 2020). Noticeable changes have been witnessed in recent decades in some South Asian countries, including India (Rai & Dimri, 2020), Bangladesh, Nepal, Pakistan (Lipczynska-Kochany, 2018), and Sri Lanka (Khaniya et al., 2019; Naveendrakumar et al., 2018; Perera et al., 2020). Various kinds of seasonal and annual precipitation and temperature changes are reported (Mondal et al., 2024). A significantly high warming trend and growing seasonal rainfall trends have been reported in various sections of South Asia. The El Niño and La Niña phenomena play a significant role in altering the Indian Ocean monsoon system, impacting regional rainfall and temperatures (Alahacoon & Edirisinghe, 2021). For instance, during El Niño events, the weakened Walker circulation affects rainfall patterns, leading to decreased rainfall in specific months (Wen et al., 2020).

As an island located in the Indian Ocean, the climate of Sri Lanka has always been affected by monsoons and similar adverse climatic conditions (Su et al., 2021). Also, variations have been observed in rainfall patterns, and a rise in temperature trends has been noted. The country has warmed by approximately 0.8 $^\circ{\rm C}$ during twentieth century, positioning it among the world’s hottest countries (Samaraweera et al., 2024). In addition, the projections of temperature and rainfall in Sri Lanka, based on the A2 and B2 scenarios using the ECHAM4 general circulation model (GCM) for downscaling, indicate that the country’s average annual temperature is expected to rise by 2.5–4.5 °C by 2080 under the A2 scenario (Marambe et al., 2015). However, diverse climate trends are exhibited by different regions within the country (De Costa, 2008). Distinct climate patterns are observed in the Western Province, a densely populated region in the wet zone, and the metropolitan area, Colombo (Perera et al., 2020). The understanding of these climate trends is considered vital in the Sri Lankan context, particularly concerning urbanization and the development of socio-economic, political, cultural, and technological methods (Wickramasinghe et al., 2023).

Literature on precipitation and temperature trends in Sri Lanka has faced limitations due to the reliance on data from a few stations. However, advancements in geospatial technologies and satellite data have significantly enhanced the capability to faithfully depict regional climate patterns. Satellite data, as exemplified by resources such as the Tropical Rainfall Measuring Mission (TRMM), Global Precipitation Climatology Center (GPCC), and Climate Hazards Group InfraRed Precipitation with Stations data (CHIRPS), present a viable solution to overcome these limitations (Rachdane et al., 2022).

Therefore, to overcome the gaps in the research, we focused on CHIRPS and Climate Hazards Group InfraRed Temperature with Stations data (CHIRTS) due to their high spatial and temporal resolutions, providing a more comprehensive understanding of the spatial variability in temperature and rainfall. The climate in Colombo has been examined in numerous studies (Perera et al., 2020; Senanayake et al., 2013), yet the temporal resolution has often been found insufficient. In addressing this, the analysis presently conducted delves into data spanning from 1980 to 2022 to discern the microclimate impact. Three specific areas are the focus of this study: the entirety of Sri Lanka, the Western Province, and the Colombo Metropolitan Area (CMA). The primary aim is to investigate potential microclimatic influences within the CMA in comparison to its surrounding areas, particularly the Western Province and the broader country. Additionally, existing relationships are ascertained by the study concerning regional climatic effects such as El Niño-Southern Oscillation (ENSO).

## Materials and methods

### Study area

**Fig. 1:**
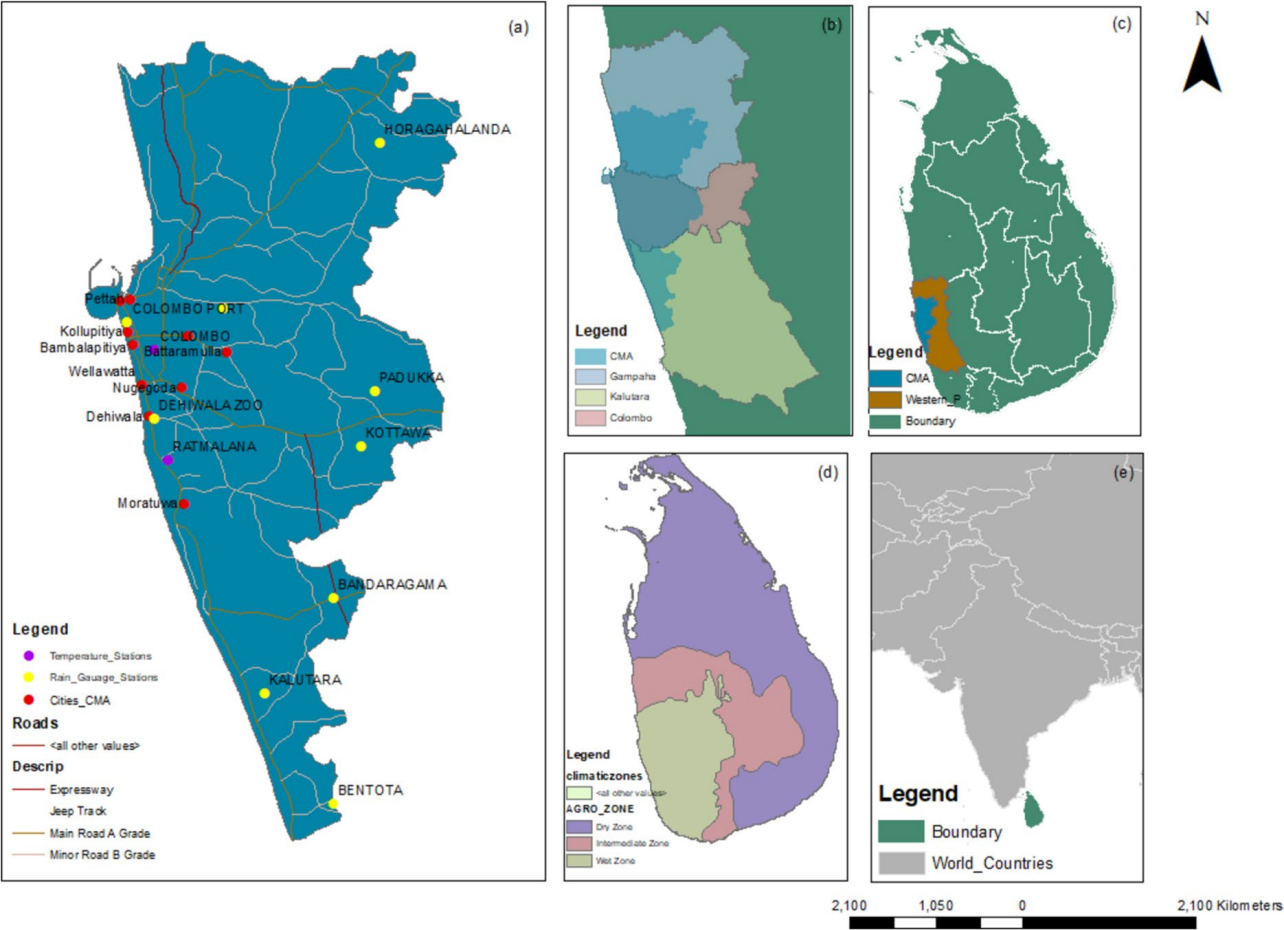
Map of the study area: **a** Colombo Metropolitan Area; **b** Western Province; **c** Sri Lanka; **d** climatic zones of Sri Lanka; **e** Sri Lanka in the Indian Ocean

As shown in Fig. 1, the CMA, which is considered the focused area for this study, is the only metropolitan region of the country (Fonseka et al., 2024). Apart from CMA, the Western Province, spanning from 30.0°N to 47.5°N and from 73.5°E to 117.5°E, is characterized by continental arid conditions, with relatively minimal influences from the East Asian Summer Monsoon. The study area experiences an annual total precipitation of approximately 2300 mm, and the average temperature is around 28 °C (Subasinghe et al., 2016).

### Meteorological data

#### Ground observations

Rain gauge measurements and temperature measurements covering the time period from 2000 to 2010 were obtained from the Meteorological Department of Sri Lanka. Monthly rainfall measurements from eight gauge stations (Angoda, Padukka, Dehiwala, Colombo Fort, Kalutara, Badaragama, Bentota, and Handapangoda) as shown in Fig. 1 were utilized to validate the rainfall products.

#### Gridded satellite products

The satellite products employed and their respective characteristics are summarized in Table 1.

**Table 1:** Gridded satellite products and characteristics

Data	Resolution	Source	Reference
CHIRPS (Climate Hazards Group InfraRed Precipitation with Station)	0.05° (5550 m)	https://www.chc.ucsb.edu/data/chirps	(Wu et al., 2019)
CHIRTS (Climate Hazards InfraRed Temperature with Station data)	0.05° (5550 m)	https://www.chc.ucsb.edu/data/chirtsmonthly	(Verdin et al., 2020)
WC climate data	~ 21 km^2^ at the equator	https://www.worldclim.org/data/worldclim21.html	(Fick & Hijmans, 2017)

### Validation of climatic data

To ensure the reliability and accuracy of the data, validation was conducted using data from stations, and all products underwent a thorough evaluation. Data from the closest grid point of each station were extracted from its corresponding gridded data product for comparison with station measurements and for point-to-pixel comparison (Zhang et al., 2018). The temperature products were validated using data from two stations, Colombo and Rathmalana, which are the only ones available within the study area. Validation was performed to assess the performance of satellite products at a specific location. The comparison metrics were computed using annual temperature and precipitation values. In this section, Pearson’s correlation coefficient is employed, as specified in Eq. (1) (Ma et al., 2021; Xu et al., 2023).

1 $$\text{Correlation }\left(r\right)= \frac{\sum_{i=1}^{n}({s}_{i}-{\upmu }_{\text{s}})({o}_{i}-{\upmu }_{\text{o}})}{\sqrt{\sum_{i=1}^{n}{({s}_{i}-{\upmu }_{\text{s}})}^{2}}\sum_{i=1}^{n}{({o}_{i}-{\upmu }_{\text{o}})}^{2}}$$

where ${s}_{i}$ represents the gridded data at time $i$, ${o}_{i}$ is the station data at time $i$, $n$ is the length of the series, ${\upmu }_{\text{s}}$ is the mean of the gridded data, and ${\upmu }_{\text{o}}$ is the mean of the station data. Scatter diagrams were used to comprehensively describe and evaluate performance. The performance was evaluated considering correlation. This criterion aligns with the World Meteorological Organization’s recommendations for computing monthly values for climate normal, aiming to prevent excessive missing data from introducing bias to the results and accounting for the interannual variability of temperature and precipitation (Nastos et al., 2016).

### Statistical analysis of climate data

To identify and quantify trends in extreme climate indices, the nonparametric Mann–Kendall (M–K) test (Mann, 1945) was utilized along with Sen’s slope estimator (Hirsch et al., 1991). These statistical approaches are well suited for detecting trends and estimating their magnitude in the computed climate indices associated with extreme weather conditions.

#### Mann–Kendall test

Among the widely used statistical techniques for analyzing trends in both hydrological and climatological time-series data, the M–K test is popular. Initially proposed by Mann in 1945 (Mann, 1945), this test has found extensive application in environmental time-series studies. The M–K test is employed, offering dual advantages. First, its nonparametric nature renders it independent of the assumption of normal distribution in the data. Second, the test demonstrates low sensitivity to abrupt interruptions in the time series due to its capability to handle inhomogeneity.

#### Sen’s slope estimator test

The determination of the magnitude of the trend within a time series is achieved through the utilization of a nonparametric technique known as Sen's estimator (Hirsch et al., 1991). Sen's nonparametric technique is applied to ascertain the true trend's slope or the annual change in amount. The positive value of Sen's slope indicates an increasing or upward trend in the time series, while its negative value signifies a decreasing or downward trend.

### Spatial analysis of climatic variables

The creation of climate indices based on monthly and annual temperature and rainfall time series, as proposed by Peterson et al. (2002), is one method used to characterize climate (Asfaw et al., 2018). This approach allows for the characterization of intensity, duration, and frequency. In this study, four major climatic indices were selected to identify and interpret the patterns of climatic variation in the study area. The selected indices were calculated using the R software interface. The examination of these various extreme climate indices was conducted in a spatial context. The analysis of time-series data, including datasets from CHIRPS and CHIRTS spanning the years 1980 to 2020, is the primary focus of our research.

#### Seasonality index

Information about inter-annual variations in seasonality can be found in the seasonality index (SI), which is produced for each year using the mean monthly rainfall (Rai & Dimri, 2020). A widely used SI, derived by Walsh and Lawler (1981), is mentioned in Eq. (2) as follows:

2 $${SI}_{i}=\frac{1}{{R}_{i}}\sum_{n=1}^{n=12}|{X}_{i,n}-\frac{{R}_{i}}{12}$$

where ${R}_{i}$ represent the total annual rainfall per year and $X$ is the actual monthly rainfall for month $n$. Table 2 showcases the physical meaning of this seasonality index.

**Table 2:** Integrated classification of climatic indices: seasonal precipitation regimes (Walsh & Lawler, 1981), rainfall anomaly intensity, and Heatwave Magnitude Index daily (HWMId)

(a)		(b)	
**Rainfall regime**	**Seasonality index**	**RAI regime**	**Classification**
Very equable	≤ 0.19	Above 4	Extremely humid
Equable but with a definite wetter season	0.20–0.39	2 to 4	Very humid
Rather seasonal with a short drier season	0.40–0.59	0 to 2	Humid
Seasonal	0.60–0.79	− 2 to 0	Dry
Markedly seasonal with a long drier season	0.80–0.99	− 4 to − 2	Very dry
		Below − 4	Extremely dry
(c)			
**Heat wave category**	**Range**		
Normal	1 ≤ HWMId ≥ 2		
Moderate	2 ≤ HWMId ≥ 3		
Severe	3 ≤ HWMId ≥ 4		
Extreme	4 ≤ HWMId ≥ 8		
Very extreme	8 ≤ HWMId ≥ 16		
Super extreme	16 ≤ HWMId ≥ 32		
Ultra-extreme	HWMId ≥ 32		

#### Rainfall anomaly index

The annual Rainfall Anomaly Index (RAI) was computed using precipitation data to examine the frequency of rainy and dry years in the study area (Aryal et al., 2022).

3 $$RAI=3\left\lfloor\frac{N-\overline N}{\overline M-\overline N}\right\rfloor;\;\text{for positive anomalies}$$

4 $$RAI=-3\left\lfloor\frac{N-\overline N}{\overline M-\overline N}\right\rfloor;\;\text{for negative anomalies}$$

where $N$ represents current monthly/annual rainfall (mm), $\overline{N }$ represents monthly/annual average rainfall of the historical series (mm), $M$ represents average of the ten highest monthly/yearly precipitations derived from the historical series (mm), and $X$ represents the average of the ten lowest monthly/yearly precipitations from the historical series (mm). In Eq. (3), positive anomalies are characterized by values above average, and in Eq. (4), negative anomalies are associated with values below normal as depicted in Table 2.

#### The Heat Wave Magnitude Index daily

One of the most sophisticated heat-wave detection measures, which takes into consideration the heatwaves’ duration and intensity, was considered in this study. Sri Lankan heatwaves from 1987 to 2016 were tracked spatiotemporally using the new Heatwave Magnitude Index daily (HWMId) (Amou et al., 2020; Russo et al., 2015). The Heatwave Magnitude Index (HWMI) has been enhanced with HWMId (Amou et al., 2020), which was created in response to some of the latter's drawbacks.

A heat wave is characterized by three consecutive days with a maximum temperature that is higher than the 90th percentile threshold (Funk et al., 2019) for the 1983–2014 reference period. The greatest intensity heat wave that happens in a particular year is referred to as "HWMId." Excess temperatures above a threshold, by summing the durations and temperature anomalies of extreme heatwave occurrences, were added. Heatwaves with different peak amplitudes and lengths that have occurred in different places and years were compared. According to Russo et al. (2015), the 90th percentile was expressed in Eq. (5) and was centered on 31-day windows.

5 $${A}_{d}= {U}_{y=1983}^{2014}{U}_{t=d-15}^{d+15}{T}_{y,t}$$

Here, the year is represented by $y$, the day is represented by $d$, and $U$ symbolizes the union of sets, with ${T}_{y,t}$ representing the daily maximum temperature on day $i$. The index was annually computed using the R programming language. In the calculation of the HWMId, corresponding duration and starting time were provided via its "hwmid" function and "extRemes" package, respectively. Still, the process can be stepped into three parts as per the overview given by Dobricic et al. (2020).

- The first step involves using Eq. (2) to determine the daily heat-wave magnitude of each day (${M}_{d}$) during a heatwave period. For a given day, (${M}_{d}$) is the normalized difference between the first and third quartile values of the time series (Russo et al., 2015).
  6 $${M}_{d}\left({T}_{d}\right)=\left\{\begin{array}{c}\frac{{T}_{d}-{T}_{30\text{y}25\text{p}}}{{T}_{30\text{y}75\text{p}}-{T}_{30\text{y}25\text{p}}}; {T}_{d}>{T}_{30\text{y}25\text{p}}\\ 0; {T}_{d}\le {T}_{30\text{y}25\text{p}}\end{array}\right.$$
  ${T}_{30\text{y}25\text{p}}$ and ${T}_{30\text{y}75\text{p}}$ are the first and third quartile values, respectively, of a 32-year time series made up of the yearly *T*_max_ values of the reference period, where ${T}_{30\text{y}}$ is the highest daily temperature on day *d* of the heat wave.
- The total heatwave magnitude for a season or year is determined by the total daily magnitudes (daily magnitudes ${M}_{d}$) of the days that make up a heatwave, constituting the magnitude of each individual heatwave within a year (*M*_hw_).
- The calculation of HWMId involves determining the cumulative daily magnitudes (represented as ${M}_{d}$) of the days comprising a heatwave, contributing to the overall magnitude of each specific heatwave within a given year (denoted as *M*_hw_).

The physical interpretation of the index is given in Table 2.

#### Mean temperature

The mean temperature is determined by calculating the average of temperature values over a specific period of time, and it can be simply calculated using Eq. (7).

7 $$\text{Mean temperature}= \frac{\text{Sum of temperatures for the decade}}{\text{Number of years in the decade}}$$

## Results and discussion

### Validation of climatic data

The accuracy of the average rainfall and temperature products was confirmed through validation using station data. Rain gauges and ground-based weather radars are two examples of conventional precipitation monitoring techniques that are still highly limited, particularly in developing nations. Other challenges include data-quality concerns and maintenance expenses (Rachdane et al., 2022). The World Meteorological Organization advises one rain gauge every 900 km^2^ in lowland areas and per 250 km^2^ in places with complex topography for all climatological reasons. Sri Lanka, being a developing nation, is still a long way from meeting these requirements (Liu et al., 2021).

**Fig. 2:**
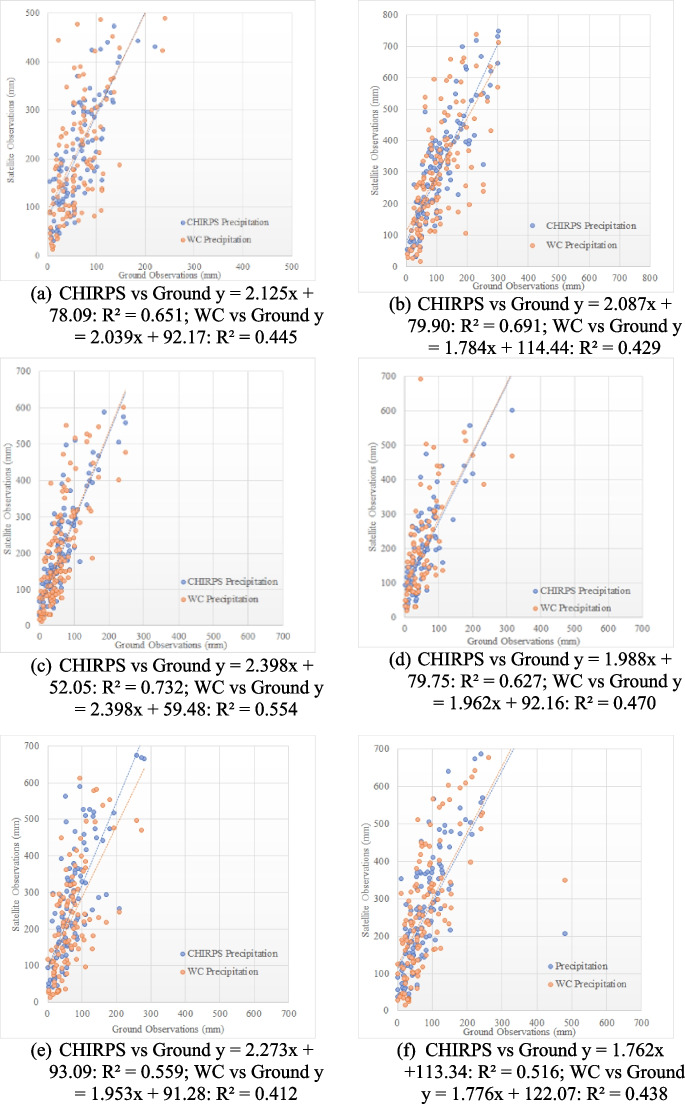

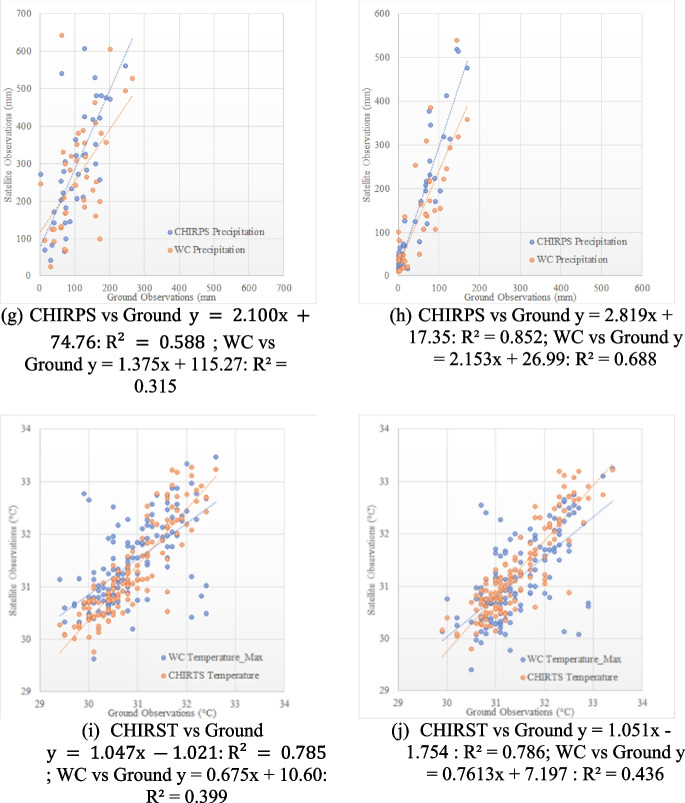
Precipitation validation for WC and CHIRPS data using ground data: **a** Angoda; **b** Padukka; **c** Dehiwala; **d** Colombo Fort; **e** Kalutara; **f** Bandaragama; **g** Bentota; **h** Hadapangoda. Temperature validation for WC and CHIRST data using ground data: **i** Colombo; **j** Rathmalana

Correlation was observed among the eight rain gauge stations (Angoda, Padukka, Dehiwala, Colombo Fort, Kalutara, Bandaragama, Bentota, Hadapangoda). There were some deviations in stations, particularly in the Hadapangala stations. These deviations could potentially be attributed to several reasons such as instrumental errors, the urban heat island effect, and local interference (Zhang et al., 2018). A thorough investigation and quality control process are deemed essential to determine the specific cause of the deviation at the station. In addition, it can be clearly seen that precipitation products have overestimated the actual precipitation levels. Regarding temperature validation, only two stations were available for this purpose, Colombo and Rathmalana, as represented in Fig. 2. However, they exhibited a strong correlation, indicating a reliable validation outcome. The correlations between WC temperature data and ground observations were lower than those of CHIRTS's and CHIRPS’s; therefore, spatial analysis in this study primarily relied on CHIRPS and CHIRTS data due to good performance (Parsons et al., 2022).

### Statistical analysis of climate data

**Fig. 3:**
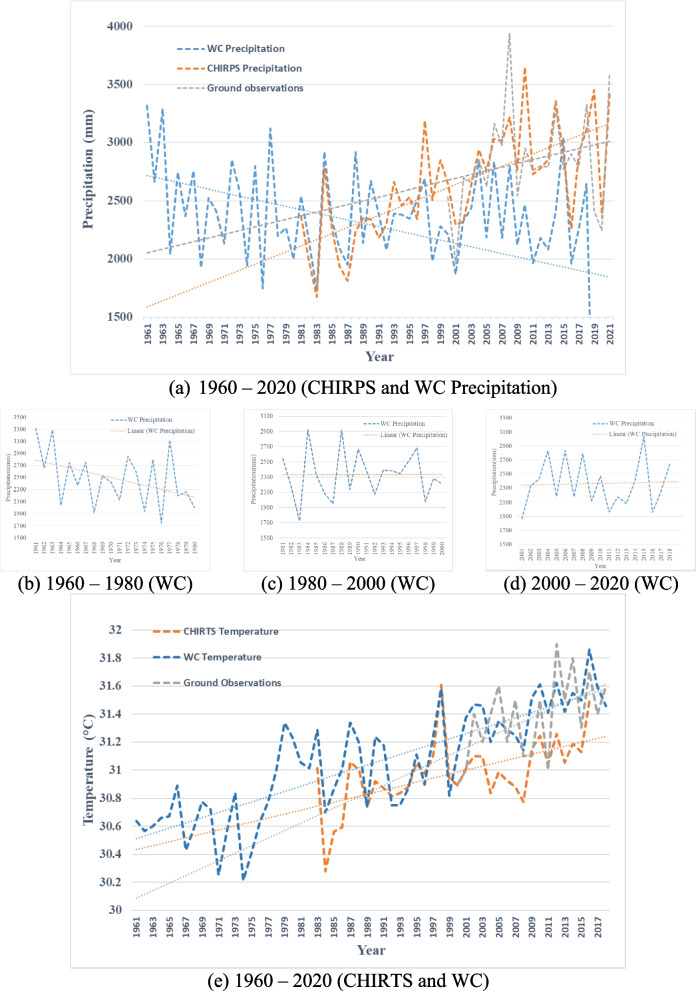
Time-series analysis of precipitation data: **a** 1960–2020 (CHIRPS and WC precipitation); **b** 1960–1980 (WC); **c** 1980–2000 (WC); **d** 2000–2020 (WC); **e** time-series analysis of temperature data

The temporal variations of temperature and precipitation were studied, and time-series graphs were produced, as shown in Fig. 3. The rainfall pattern for the period from 1960 to 2020 was reproduced by using both CHIRPS and WC gridded precipitation data. A unimodal rainfall pattern was observed in both CHIRPS and WC precipitation data for the study area, and both datasets indicated an increasing rainfall trend from 1980 to 2020. However, the overall rainfall pattern from WC data starting in 1960 shows a decreasing trend, leading to the conclusion that the decades from 1960 to 1980 had the most significant impact on decreasing rainfall.

These results are consistent with the findings of Alahacoon and Edirisinghe (2021), indicating a tendency of increased rainfall from 1980 onwards. It was further confirmed that when considering average rainfall throughout history, the highest amount of rainfall was recorded in the years 2010, 2011, and 2014. In the context of Karunathilaka et al. (2017), which used 50 years of data to examine precipitation patterns, the WC data indicates a declining trend in rainfall. However, given that Sri Lanka is a tropical country influenced by various climatic systems, the changes in rainfall on a decadal basis need to be studied in conjunction with external factors and anthropogenic impacts.

Similarly, a comparison was made between the CHIRST and WC temperature data, as depicted in Fig. 5. Trends of increasing annual mean maximum temperatures every 50 years are evident in the WC data, while rising trends in mean maximum temperatures starting from 1983 are denoted by CHIRTS. In contrast to precipitation, the rate of temperature change is significant from 1980 to 2020. Similar to the WC data, the notable trends in CHIRTS are reasonably constant between 1980 and 2020. On a monthly scale, Sen’s slope and M–K tests were employed to identify patterns in the temperature and precipitation data over several decades. The outcomes are presented in Table 3, demonstrating how the two tests performed.

**Table 3:** Summary of Mann–Kendall and Sen’s slope tests

Variable	Region	Decade	Kendall's tau	*P* value	Sen’s slope
Temperature	Sri Lanka	1980–1990	0.057	0.447	0.001
		2000–2010	− 0.072	0.251	− 0.001
		1990–2000	0.014	0.829	0.000
		2010–2020	0.076	0.306	0.002
	Colombo	1980–1990	0.025	0.737	0.001
		1990–2000	− 0.017	0.791	0.000
		2000–2010	− 0.043	0.485	− 0.001
		2010–2020	0.024	0.754	0.001
Precipitation	Colombo	1980–1990	0.027	0.681	0.049
		1990–2000	0.073	0.239	0.171
		2000–2010	0.079	0.227	0.222
		2010–2020	− 0.036	0.546	− 0.082
	Sri Lanka	1980–1990	0.024	0.712	0.056
		1990–2000	0.057	0.361	0.125
		2000–2010	0.041	0.533	0.097
		2010–2020	− 0.025	0.349	− 0.008

For temperature, decreasing *P* values from 1980–1990 to 2010–2020, as depicted in Table 3, suggest a strengthening indication of a trend in the climate data. The decreasing *P* values imply that there is increasing statistical evidence against the null hypothesis, assuming no trend, which may suggest a substantial trend in climate data for both Colombo and Sri Lanka over the specified time periods. In summary, for Sri Lanka, there are minor positive and negative trends indicating subtle changes in the climate data. In contrast, Colombo exhibits consistent positive trends with small to moderate magnitudes, indicating gradual increases in the climate variable over the specified decades. These trends, while present, might not be significant enough to have substantial impacts on the climate in the short term. Furthermore, the meteorology department of Sri Lanka confirms that the country experiences temperature variations due to its latitude (Samaraweera et al., 2024).

As depicted in Table 3 for precipitation, both Colombo and Sri Lanka exhibit strengthening evidence of climate trends, with the strongest evidence observed in the third periods for both regions. These results suggest a significant and increasing trend in the precipitation data, especially during the third periods considered. However, this confirms the fact that despite the temperature variations, the country’s climate is mainly influenced by precipitation.

### Spatial analysis of climatic variables

Spatial analysis of data involved the calculation of indices for all gridded datasets. Due to the coarser resolution of the WC data, it was not utilized further in this spatial analysis, as the CMA has not exhibited proper variability in the data. The indices were calculated using CHIRPS and CHIRTS data for the study area from 1980, and summaries of 10-year periods were presented. The year-to-year fluctuations could be reduced by calculating the decadal average of rainfall and temperatures, which also demonstrated a similar trend over time (De Costa, 2008).

**Fig. 4:**
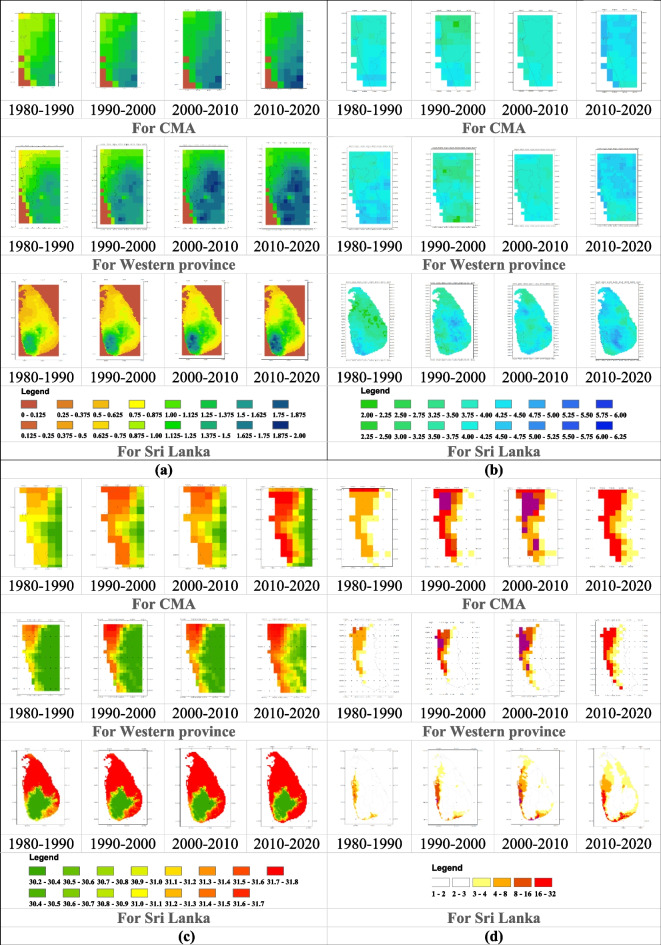
**a** Rainfall seasonality index; **b** rainfall anomaly index from CHIRPS; **c** mean temperature of the study area from 1983 to 2018; **d** heat wave magnitude index of the study area from 1983 to 2018

When conducting rainfall seasonality index analysis for the study area from 1980, summaries of 10-year periods were presented, as shown in Fig. 4. The change in seasonality is becoming more pronounced, as indicated in Fig. 4, with the most significant rate of change occurring in the Western Province in contrast to other regions of the country. While the seasonality of the country changes from 1.17 to 1.45 over decades, as shown in Table 4, the seasonality of CMA changed from 1.25 to 1.83 on average over the decades, with the southern part of the CMA experiencing a more significant increase. Seasonality is expanded from inland to coastal area. As depicted in Table 4, both CMA and Western Province are in the extreme region from the first decade, and similarly, the country falls into that regime in the 2000–2010 period.

**Table 4:** Results of climatic index calculation

		1980–1990	1990–2000	2000–2010	2010–2020
**HWMId**	CMA	20.375	36.458	42.278	43.444
	Western	19.566	30.016	37.336	37.705
	Sri Lanka	11.765	15.077	19.015	20.701
**MT**	CMA	30.464	31.106	31.145	31.252
	Western	30.213	30.788	30.441	30.522
	Sri Lanka	30.893	31.166	31.260	31.410
**RAI**	CMA	4.550	4.020	4.260	5.350
	Western	4.920	3.770	4.430	5.300
	Sri Lanka	3.660	4.370	4.550	5.060
**Seasonality**	CMA	1.250	1.570	1.82	1.830
	Western	1.380	1.700	1.970	1.990
	Sri Lanka	1.170	1.290	1.420	1.450

Next, the rainfall anomaly index was calculated for the entire Sri Lanka, Western Province, and CMA, as shown in Fig. 4. At the country level, high anomaly values can be observed in different parts of the country over the four decades. While the country’s rainfall anomaly on average changes from 3.66 to 5.06, becoming an extremely humid country with reference to Table 2 over decades, the CMA’s rainfall anomaly changes from 4.55 to 5.35 on average over the decades. In literature, there are several studies which showcase similar results (Alahacoon & Edirisinghe, 2021; Naveendrakumar et al., 2018; Perera et al., 2020) that discuss the increasing pattern of rainfall in the country. CMA is classified as an extremely humid regime from 1980, and it remains in the same region of classification. In the Western Province and CMA, compared to other decades, the period from 2010 to 2020 can be identified as the most extremely humid period (Naveendrakumar et al., 2018).

In the context of rainfall, a clear trend is observable: wetter regions are becoming even wetter, while drier areas are progressively drying out over time (Imteaz & Hossain, 2023). Rainfall seasonality stands as one of the primary factors contributing to this climate change phenomenon. Furthermore, variations in monsoon patterns and climatic systems like El Niño and La Niña can also influence seasonality, alongside changes in land use (De Costa, 2008; Naveendrakumar et al., 2018). Other than that, the emerging global warming and subsequent climate change can impact this; for example, due to the climate change, earth’s atmospheric conditions are changing, and as a result, meteorological events are increasing. This influences the annual rainfall which alters the precipitation distribution in seasonality and rainfall anomaly (Imteaz & Hossain, 2023).

To study climatic variability, surface temperature plays a crucial role in identifying patterns of change. Mean temperatures from CHIRTS, as shown in Fig. 4, were analyzed to identify the temperature pattern in Sri Lanka. As depicted in Table 4, temperature values at the country, province, and CMA levels are increasing. Coastal temperature is higher and then it moves toward inland while following the urban pattern (Naveendrakumar et al., 2018). However, it can be observed that the mean temperature decreased from 1990 to 2000 and then showed an increasing pattern. Particularly in the Western Province and CMA, coastal temperatures underwent dramatic changes from 1980 to 2020 (Zubair et al., 2008). Overall, mean surface temperatures increased at a rate of 0.05 °C per year for the country and 0.12 °C for CMA during a 35-year period (1983 to 2018).

One of the significant reasons for the observed warming trend is identified as the local heat island effect. This effect is mainly caused by two factors: increasing urbanization (Chatterjee & Majumdar, 2022) and the escalating impact of greenhouse gases (Singh & Sharston, 2022). However, CMA reveals higher temperature values, and similar results were reported by Ranagalage et al. (2017). In 10-year periods from 1981 to 2018, the maximum values of the HWMId from CHIRTS observations for average temperature are depicted in Fig. 4.

The yearly records and patterns of the most severe heat wave events are illustrated in Fig. 4. It is indicated that heat wave events from 11.765 (Very extreme) to 20.701 (Super extreme) have occurred across Sri Lanka. Since Sri Lanka is closer to the equator, the country poses a highest risk of heave wave (Samaraweera et al., 2024). For example, the International Water Management Institute estimates that between 2001 and 2013, 23% of Sri Lanka's population was exposed to dangerous heatwaves (Amarnath et al., 2017). On the other hand, the magnitude of heat-wave events in CMA varied from Super extremes to Ultra extreme with reference to Table 2. The western and southern parts of the country, including CMA and Western Province, experienced the most significant impact. The above specific regions have not experienced any (zero) to extreme heat wave events, despite the fact that other parts of the country have higher mean temperatures and are typically hit by high temperatures. It was also evident that the heat waves of the past several years were more severe in terms of their size and geographic reach.

Elevated Land Surface Temperature (LST) values were predominantly observed near the coastline belt or the more urbanized area of the CMA, as depicted in Fig. 4, and the relationship between heat island effect and increase of temperature cities needs to be studied further (Naveendrakumar et al., 2018). On the other hand, locations with high LST had significantly increased as they approached the CMA's interior, conforming to the region's spatial trend of urban growth.

**Fig. 5:**
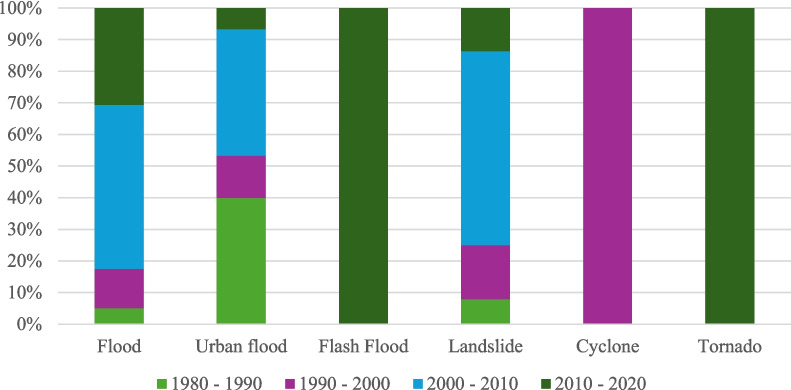
Extreme climatic events in CMA

Different geographical scales are represented by Colombo and Sri Lanka, with Colombo being the largest city and commercial capital of Sri Lanka. When discussing climate differences between Colombo (a city) and Sri Lanka (an entire country), it is important to consider that climate can vary significantly within a country due to geographical features such as mountains, coastal areas, and plains (De Costa, 2008; Rathnayake, 2019). While both Colombo and the rest of Sri Lanka share a tropical climate, there can be microclimatic differences within the country (Alahacoon & Edirisinghe, 2021). Considering all the abovementioned indexes and the trend analysis, it was shown that CMA has exhibited a different climatic pattern when compared with the country as a whole (Naveendrakumar et al., 2018). The findings of this study indicate that the region experienced microclimate changes, which might be mostly due to urbanization. To identify the climatic pattern of Colombo, the Disaster Management Centre summarized the intensity and frequency of observed extreme climatic events, as detailed below in Fig. 5. The extreme events were only considered for the Colombo Metropolitan Area.

In Fig. 5, it can be further confirmed that both tornadoes, flash floods, and floods were more frequent in the decade from 2010 to 2020. The dynamic trends in rainfall extremes in Sri Lanka have shown that Colombo experienced the highest value in 2010 among all severe extremes in the historical records (Naveendrakumar et al., 2018). It is important to note that the connection between climate change and the factors contributing to flash floods is influenced by a combination of local weather conditions, topography, land use, and infrastructure (De Costa, 2008). Therefore, attributing a single flash flood event directly to climate change can be challenging. However, scientific studies often find that heavy rainfall events are becoming more frequent and more intense in some regions, which is consistent with what would be expected in a warming climate. In the first 5 years of the twenty-first century, 2 years of severe drought and one major flood event were faced by Sri Lanka, as highlighted by Eriyagama et al. (2010), particularly in the districts of Ratnapura and Kalutara, which typically experience flooding once or twice a year.

Other than floods, landslides are also triggered by rainfall. However, the frequency of cyclones has decreased during the twentieth century (Samaraweera et al., 2024) despite the increase in tropical cyclone intensity in the Bay of Bengal (Balaguru et al., 2014). This highlights the critical need for measures to study climate change.

### Impact of teleconnections

Research has demonstrated that teleconnections, specifically ENSO and Indian Ocean Dipole (IOD), are significant drivers of rainfall patterns in Sri Lanka. Wijeratne et al. (2022) conducted an extensive examination of the relationship between ENSO and IOD indices and extreme hydrological events. During the El Niño phase, Colombo experienced a negative and statistically significant correlation with the Rainfall Anomaly Index (RAI) at the 0.05 significance level. De Costa (2008) similarly noted that post–El Niño years generally resulted in negative annual rainfall anomalies across Sri Lanka. However, Colombo and Ratnapura were exceptions, suggesting that while ENSO significantly impacts the broader region, local factors such as urbanization and topography modify these effects, particularly in urbanized areas like Colombo (De Costa, 2008).

Urbanization has had a profound impact on the local climate in Colombo, especially concerning temperature. The urban heat island effect, where urban areas experience higher temperatures than their rural counterparts, is well documented globally (Yang & Zhao, 2024), and Colombo is no exception. As the city undergoes rapid development, with significant reductions in forest cover and increases in impervious surfaces such as concrete and asphalt, localized temperature increases have become more evident (Manawadu & Wijeratne, 2021; Wijeratne et al., 2022). The rapid urbanization of Colombo, marked by human-made changes in land use, has contributed to significant temperature rises, particularly during dry spells associated with El Niño, resulting in more frequent extreme weather events like heatwaves.

While urbanization is a key factor driving local temperature increases, global-scale processes like ENSO also influence temperature extremes in Colombo. Studies have shown that during El Niño phases, Colombo experiences not only a reduction in rainfall but also significant temperature increases, which exacerbate the urban heat island effect (De Costa, 2008; Wijeratne et al., 2022). Naveendrakumar et al. (2018) further highlighted that wet-zone cities, including Colombo, are profoundly affected by temperature extremes, particularly during ENSO-related droughts. Malmgren et al. (2007) revealed that cities such as Colombo, Jaffna, and Puttalam exhibit greater temperature and precipitation variability during El Niño compared to La Niña years. However, neutral years also showed similar temperature patterns to El Niño periods, underscoring the role of local factors such as urbanization in modulating temperature variations.

The interaction between urbanization and teleconnections reveals complex climate dynamics in Colombo. While ENSO and IOD significantly influence rainfall and temperature patterns on a global scale, rapid urbanization of Colombo has introduced localized temperature extremes that deviate from broader trends. The reduction of vegetative cover and the expansion of urban infrastructure have amplified the urban heat island effect, leading to prolonged periods of higher-than-average temperatures (Manawadu & Wijeratne, 2021; Wijeratne et al., 2022).

## Discussion

The main objective of this study is to investigate the long-term variability of temperature and rainfall in the study area and to identify any climate change indicators. The statistical metrics between CHIRPS and ground observations suggest that satellite products can be effectively used in analysis, though they require validation with ground observations. Satellite grid products have proven useful for spatial and temporal analysis in Sri Lanka, where spatial data is limited. The long-term analysis indicates that both temperature and rainfall are increasing over time.

Spatial analysis includes seasonality indices, rainfall anomalies, mean temperature, and heatwave magnitude indices. Decadal and monthly rainfall data over study area exhibit variability and uncertainty. Detecting trends in climate data is crucial for understanding and developing adaptation actions at regional or local scales. The results show that the Colombo Metropolitan Area (CMA) experiences different climate patterns compared to Sri Lanka as a whole, with increased temperatures and decreased rainfall. As an island in the Indian Ocean, the country has a low-lying coastal belt, with one third of the population living along the coast. Therefore, settlement and infrastructure are affected by the climate and vice versa. Similarly, being coastal, the only metropolitan (CMA) has been significantly influenced by this phenomenon. Notable changes in the study area, particularly the rise in flooding occurrences, are linked to significant urbanization and human activities in the CMA (Fonseka et al., 2024). These findings underscore the importance of considering human-induced factors, such as urbanization, in discussions about climate change and its local impacts.

Over the past few decades, numerous studies have investigated rainfall and temperature variability on global, regional, and subregional scales (Fathian et al., 2020; Nkemelang et al., 2018; Perera et al., 2020; Ranagalage et al., 2017; Rathnayake, 2019). Sri Lanka is an important research destination for this climate analysis. Distinct climate patterns are observed in the Western Province, a densely populated region in the wet zone, and the CMA (Subasinghe et al., 2016). Understanding these climate trends is vital for Sri Lanka, particularly regarding urbanization and the development of socio-economic, political, cultural, and technological strategies (Wickramasinghe et al., 2023). Despite numerous studies on climate of Colombo (Fonseka et al., 2024; Perera et al., 2020; Senanayake et al., 2013; Subasinghe et al., 2016), temporal resolution has often been insufficient. This study addresses this gap by analyzing data from 1980 to 2022 to assess microclimate impacts.

Comprehensive analysis of rainfall variability and trends using historical time series is crucial for designing and implementing national policy planning to enhance climate resilience. Assessments of changes in historical trends regarding averages and extremes are essential for introducing adaptation policies to prepare for future extreme events in Sri Lanka. Analyzing extreme rainfall and temperature is also critical for developing policies related to long-term planning in agriculture, national heritage sites, tourism, disaster resilience, and other sectors directly impacted by weather events. As an agricultural country, Sri Lanka can benefit from trend and extreme climate studies to introduce appropriate cropping patterns and develop new varieties that can withstand extreme temperatures.

## Conclusions

This study emphasizes how monthly CHIRPS and CHIRTS data can be suitably utilized in the replacement of station rainfall and temperature data in precipitation and temperature trend analysis, thereby accurately reflecting the impact of climate variability in Sri Lanka. While statistically conclusive evidence may be lacking, changes over time have been exhibited in both the variability and magnitude of rainfall and temperature.

The biggest obstacle to quantifying extreme indices in Sri Lanka is typically the lack of long-term, regionally dispersed, high-quality climate data appropriate for the study of extremes. Because there are few and no gaps in the station data, it has been shown in this study that satellite data can accurately reflect the current climate of Colombo over an extended period of time.

These findings underscore the importance of comprehending and addressing the impacts of climate change on temperature extremes to mitigate potential adverse impacts on human activities and the environment. Understanding the specific reasons for spatial changes in rainfall anomalies often necessitates extensive climate modeling and data analysis.

## Data Availability

No datasets were generated or analysed during the current study.

## Declarations

All authors have read, understood, and have complied as applicable with the statement on “Ethical responsibilities of Authors.”
